# Beckwith-Wiedemann syndrome

**DOI:** 10.11604/pamj.2023.45.17.38741

**Published:** 2023-05-05

**Authors:** Deeksha Mishra, Vivek Chakole

**Affiliations:** 1 Department of Anaesthesia, Jawaharlal Nehru Medical College, Datta Meghe Institute of Medical Sciences, Sawangi, India

**Keywords:** Beckwith Wiedemann syndrome, macroglossia, nasal intubation

## Image in medicine

A female infant aged 2-years, weighed 15 kg was brought to the outpatient department (OPD) with the chief complaint of difficulty in speech. She had a normal vaginal delivery at full term, weighing 3 kg, with no noteworthy family history. The infant had a device closure procedure after being diagnosed with patent-ductus-arteriosus at birth. Her syndromic face, macroglossia, and high arched palate were discovered during the examination, leading to a clinical diagnosis of Beckwith-Wiedemann Syndrome. A 9.6 g/dl hemoglobin level, elevated alpha-fetoprotein levels, and normal liver and renal function tests were observed in the recent investigations. The child was scheduled for a median glossectomy for which we chose to perform nasal intubation. Prior to surgery, the child was kept nil by mouth (2 hours for water and 6 hours feed). Zero point forty-five percent (0.45%) dextrose-normal saline was administered as the maintenance fluid. A cart was maintained on hand for difficult airways that contained a laryngeal mask airway, various sized face masks, a nasopharyngeal airway and a bougie. Induction was initiated with injection ketamine and inhalation sevofluorane after premedication with injection glycopyrrolate and injection fentanyl. By inserting a nasopharyngeal airway, the initial difficulty in mask ventilation was resolved, which also helped us in checking the patency of the nostril. Finally, a 4.5 flexometallic tube was inserted through the right nostril, guided through the vocal cords with the help of Mc-Gills forceps. The position of the tube was confirmed through auscultation and capnograph. After the procedure, she was reversed and shifted to pediatric intensive care unit (PICU) with an endotracheal tube in situ to prevent post-operative tongue fall.

**Figure 1:**
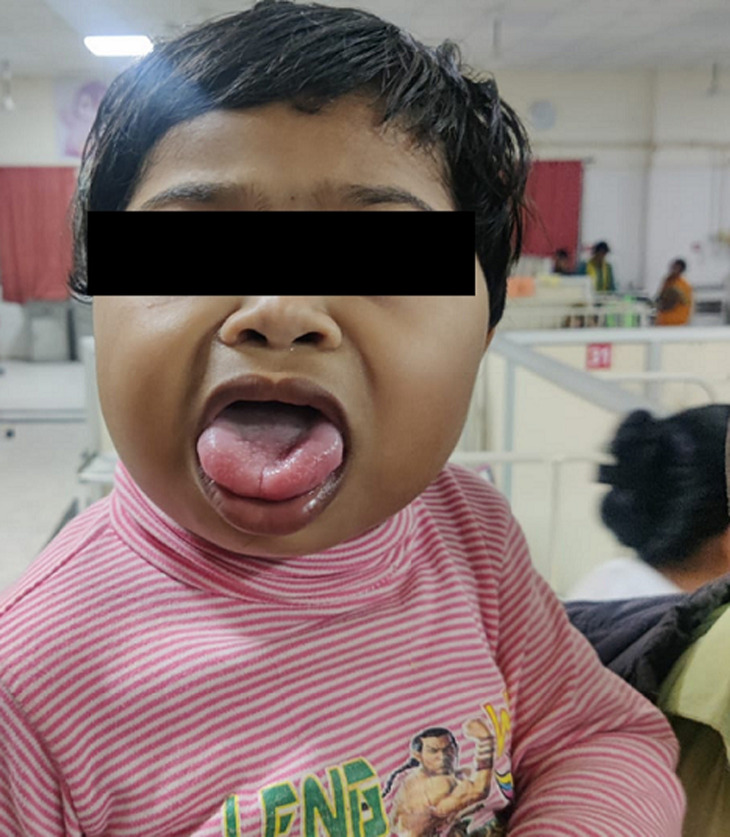
pre-operative image of the child Beckwith-Wiedemann syndrome

